# Glucagon-Like Peptide-1 (GLP-1) Agents and Cardiovascular Risk in Non-diabetic Patients: A Descriptive Analysis of National Health and Nutrition Examination Survey (NHANES), 2011–2018

**DOI:** 10.7759/cureus.94373

**Published:** 2025-10-12

**Authors:** Fatimot Disu, Nkiruka L Okoro, Adaora W Mochu, Andrew R Martey, Salihu Shittu, Joshua T Green, Williams C Azubike

**Affiliations:** 1 Stroke/Neuroscience, University Hospital Southampton NHS Foundation Trust, Southampton, GBR; 2 Pediatrics, Garki Hospital Abuja, Abuja, NGA; 3 Oncology, Garki Hospital Abuja, Abuja, NGA; 4 Family Medicine, Jonelta Foundation School of Medicine, University of Perpetual Help System Dalta, Manila, PHL; 5 Surgery/Orthopaedics, Korle Bu Teaching Hospital, Accra, GHA; 6 Internal Medicine, Oasis Kidney Care Center, Houston, USA; 7 Surgery, Sibley Memorial Hospital, Washington DC, USA; 8 Endocrinology, Endo Pharmaceuticals, Lawrenceville, USA

**Keywords:** all-cause mortality, cardiovascular mortality, glp-1 receptor agonists, nhanes, non-diabetic adults, obesity

## Abstract

Background: Glucagon-like peptide-1 receptor agonists (GLP-1 RAs) have demonstrated cardiovascular benefit in patients with type 2 diabetes and, more recently, in obese adults without diabetes. However, real-world uptake of these agents during their early introduction remains unclear. The objective of this study was to describe the prevalence of GLP-1 RA use and provide baseline, pre-uptake, population-level estimates of major adverse cardiovascular events (MACE, including myocardial infarction, stroke, and cardiovascular death) and mortality among obese adults without diabetes, in the United States (US), using pooled National Health and Nutrition Examination Survey (NHANES) 2011-2018 data.

Methods: We analyzed adults aged ≥20 years with measured obesity (BMI ≥30 kg/m²) who did not meet criteria for diabetes in NHANES 2011-2018 (unweighted n = 6,065, representing a weighted US population of ≈69 million). Medication use was ascertained from the prescription drug questionnaire (medications taken in the prior 30 days). Baseline demographics, anthropometrics, and cardiometabolic risk factors are reported as survey-weighted estimates. Mortality (all-cause and cardiovascular) was ascertained via linkage to the 2019 National Death Index.

Results: Among 6,065 obese, non-diabetic adults, none reported GLP-1 RA use in the 30 days preceding survey participation. The mean age was 45.9 years, and the mean body mass index was 35.7 kg/m². The cohort included 37,127,528 (53.6%) female and 44,259,809 (63.9%) non-Hispanic White participants. Hypertension was reported by 25,745,249 (37.2%), and 29,749,000 (42.9%) had smoked ≥100 cigarettes. Over follow-up, 2,661,169 (3.8%) of participants died from any cause, and 786,635 (1.1%) from cardiovascular causes. Because no GLP-1 RA users were identified, comparative (adjusted odds) analyses were not possible.

Conclusion: In NHANES 2011-2018, GLP-1 RA use among obese US adults without diabetes was absent, reflecting minimal community uptake in this period. These survey-weighted estimates provide baseline, pre-uptake cardiovascular and mortality benchmarks and emphasize the need for analyses using more recent data to evaluate real-world GLP-1 adoption and outcomes.

## Introduction

One of the most significant health problems in the United States (US) pertains to obesity, and at present, nearly 42% of adults in the United States are obese [1]. Obesity is closely linked to negative cardiometabolic health, such as hypertension, dyslipidemia, insulin resistance, and elevated vulnerability to major adverse cardiovascular events (MACE) like myocardial infarction, stroke, and cardiovascular mortality [2]. Although type 2 diabetes mellitus has conventionally been established as a major risk factor for cardiovascular morbidity and mortality, recent findings demonstrate that obesity alone, regardless of diabetes, will also play a significant role in cardiovascular disease (CVD) burden [3-4]. Therefore, interventions to reduce cardiovascular risk among obese, non-diabetic people remain an important area of investigation [5].

Over the past few years, glucagon-like peptide-1 receptor agonists (GLP-1 RAs) have revolutionized the management of type 2 diabetes and recently have emerged as a promising therapeutic option for obesity [6]. Formally intended to manage glycemia, GLP-1 RAs (liraglutide, semaglutide, and dulaglutide) have shown considerable weight loss when they partially impair gastric emptying, decrease food awareness, and increase resolution [7-8]. In addition to weight loss and metabolic control, an impressive number of randomized controlled trials and meta-analyses have demonstrated a cardiovascular protective effect of GLP-1 RAs in populations with diabetes, hence, lowering risks of myocardial infarction, stroke, and cardiovascular mortality [9-10]. These findings have resulted in their inclusion in cardiovascular risk reduction recommendations in patients with diabetes and established CVD [11].

However, less is established and known about the cardiovascular effects of GLP-1 RAs in obese, non-diabetic adults [12]. Although weight reduction itself is likely to reduce cardiovascular risk, it is not known whether GLP-1 RAs have any independent cardioprotective effects in non-diabetic patients, or whether these effects may largely depend on improvements in weight and blood pressure, as well as cardiovascular lipid burden [13-14]. These issues are being examined in ongoing clinical trials, but evidence from large, population-based studies of non-diabetic users remains limited [15]. With the rapid increase in the use of GLP-1 RAs for weight management among non-diabetic adults, particularly higher dose formulations such as semaglutide 2.4 mg that are approved for obesity treatment, the cardiovascular implications of this therapeutic shift are highly relevant in both clinical and population health contexts [16-17].

In this study, National Health and Nutrition Examination Survey (NHANES) data were utilized to assess the cardiovascular risk in non-diabetic patients on GLP-1 agonists. As a nationally representative survey of the US population, NHANES collects comprehensive data on demographics, health status, medication use, and cardiovascular outcomes [18]. By pooling multiple NHANES cycles, researchers can achieve a sufficient sample size to evaluate relatively uncommon exposures and outcomes, including GLP-1 RA use, among obese, non-diabetic adults [19]. Examining the prevalence and odds of major adverse cardiovascular events (MACE) among this population can help clarify whether GLP-1 RAs provide cardiovascular protection outside of diabetic contexts, thereby informing clinical practice and future guideline development [20].

The main objective of the study was to describe the prevalence of GLP-1 receptor agonist use and to provide baseline, pre-uptake, population-level estimates of major adverse cardiovascular events (myocardial infarction, stroke, or cardiovascular death) and mortality among obese US adults without diabetes using pooled NHANES data from 2011 to 2018. Given the early time frame of GLP-1 RA adoption represented by these cycles, the study is intentionally descriptive and aims to establish pre-uptake benchmarks rather than perform comparative inference between users and non-users. This study aims to deepen existing knowledge in the cardiovascular context before the widespread use of GLP-1 RA, and to inform future population-based evaluations of its real-world effectiveness by providing these baseline estimates.

## Materials and methods

Study design and data sources

This study used data from the National Health and Nutrition Examination Survey, a nationally representative program administered by the National Center for Health Statistics (NCHS) and the Centers for Disease Control and Prevention (CDC) [21]. NHANES employs a multistage, stratified probability sampling design to capture the health and nutritional status of the civilian, noninstitutionalized US population. Data are collected through structured household interviews, standardized physical examinations, and laboratory testing.

For this analysis, four consecutive NHANES cycles (2011-2018) were pooled. Pooling increases statistical power and stability of estimates, particularly for exposures or conditions with low prevalence. Mortality status was determined through linkage of NHANES participants to the 2019 National Death Index (NDI) public-use linked mortality files, which provide follow-up till December 31, 2019 [22].

Study population

The analytic sample was restricted to adults aged ≥20 years with obesity, defined as body mass index (BMI) ≥30 kg/m², based on measured height and weight obtained in the examination component. Participants with diabetes were excluded in order to focus on the research question regarding GLP-1 receptor agonist use in non-diabetic populations. Diabetes was defined as self-reported physician diagnosis, current use of non-GLP-1 antidiabetic medications, and glycated hemoglobin (HbA1c) ≥6.5%.

After applying these criteria, the final non-diabetic obese analytic sample included 6,065 participants. Within this group, no individuals reported current GLP-1 receptor agonist use. Consequently, analyses were descriptive only, with no regression modeling possible. As a result, the original comparative objective (estimating odds of MACE among GLP-1 RA users versus non-users) could not be evaluated; the study aim was therefore to provide descriptive, population-level, pre-uptake estimates of GLP-1 RA prevalence, MACE prevalence, and weighted mortality benchmarks in obese, non-diabetic US adults.

Exposure and outcome definitions

The planned exposure was the current use of a GLP-1 receptor agonist, identified in the prescription drug questionnaire, which records all prescription medications taken in the past 30 days. Agents of interest included liraglutide, semaglutide, and dulaglutide. For completeness, the exposure definition is retained here, but because no participants reported GLP-1 RA use in the analytic sample, the exposed group was empty and exposure-outcome modeling was not possible.

The primary outcome of interest was major adverse cardiovascular events, defined as the composite of (a) self-reported physician diagnosis of myocardial infarction, (b) self-reported physician diagnosis of stroke, or (c) cardiovascular mortality. Mortality outcomes were determined from NDI linkage. Cardiovascular deaths were classified using International Statistical Classification of Diseases and Related Health Problems, 10th Revision (ICD-10) codes I00-I99 [23]. All-cause mortality was examined as a secondary endpoint.

Participant characteristics

We extracted demographic, socioeconomic, lifestyle, and clinical information from NHANES to describe the study population. Demographic variables included age, sex, and race/ethnicity (non-Hispanic White, non-Hispanic Black, Hispanic, or Other). Socioeconomic indicators included educational attainment and family poverty income ratio. Lifestyle factors included smoking status (≥100 cigarettes over a lifetime) and self-reported moderate-to-vigorous physical activity. Clinical characteristics included measured BMI, direct high-density lipoprotein (HDL) cholesterol, total cholesterol, self-reported hypertension, and physician-diagnosed diabetes. Mortality outcomes (all-cause and cardiovascular-specific) were ascertained through linkage to the National Death Index.

Missing data

We evaluated the extent of missing data for all key analytic variables. The proportion of missing values was minimal: BMI, 3.1; HDL cholesterol, 1.3%; and total cholesterol, 1.3%. Age, all-cause mortality, and cardiovascular mortality had no missing data. Because NHANES employs a complex survey design and specialized survey weights, multiple imputation was not feasible. Analyses were therefore performed on complete cases.

Statistical analysis

Analyses were conducted using Stata/SE version 18.0 (StataCorp, College Station, USA). All procedures incorporated NHANES survey weights, primary sampling units, and strata, ensuring estimates were representative of the adult US population.

Given the absence of GLP-1 use among non-diabetic obese participants, analyses were limited to descriptive statistics. Weighted means and standard errors were calculated for continuous variables, while categorical variables were expressed as weighted percentages. Mortality outcomes (all-cause and cardiovascular) were summarized using weighted prevalence estimates. Kaplan-Meier survival curves were generated to illustrate cumulative mortality over follow-up. All analyses, therefore, focused on descriptive estimation of prevalence and weighted mortality benchmarks for the pre-uptake era; no inferential comparison or regression modeling between GLP-1 users and non-users was performed because the exposed group was empty.

Regression modeling was not performed due to the lack of variation in the exposure variable (GLP-1 receptor agonist use). This limitation is reported transparently as a finding of the study.

Ethical considerations

This study was conducted using publicly available, de-identified NHANES data. The NHANES protocol is reviewed and approved by the NCHS Research Ethics Review Board, and all participants provided written informed consent at the time of data collection. Because this project involved secondary analysis of anonymized data, additional institutional review board approval was not required. All analyses were performed in accordance with the ethical principles of the Declaration of Helsinki, with careful attention to maintaining confidentiality and the responsible use of participant data to advance public health research.

## Results

The weighted baseline characteristics of obese, non-diabetic adults in the United States, from NHANES 2011-2018 data, are presented in Table 1. The analytic sample included 6,065 participants, representing a nationally weighted population of more than 69 million adults.

**Table 1:** Baseline characteristics of obese, non-diabetic US adults (NHANES 2011–2018) Data are survey-weighted estimates from National Health and Nutrition Examination Survey (NHANES), 2011–2018. Continuous variables are presented as weighted means±standard deviations. Categorical variables are reported as weighted counts (n) with percentages. Mortality outcomes are based on linked National Death Index (NDI) follow-up till 2019.

Characteristic	Weighted mean (SD) or %
Age (years), mean±SD	45.91±15.78
Body mass index (kg/m^2^), mean±SD	35.72±5.60
Direct HDL-cholesterol (mg/dL), mean±SD	49.29±13.57
Total cholesterol (mg/dL), mean±SD	195.03±38.90
Family poverty income ratio, mean±SD	2.90±1.64
Gender, %	-
Male	32,151,114 (46.4%)
Female	37,127,528 (53.6%)
Race/ethnicity, %	-
Mexican American	7,560,130 (10.9%)
Other Hispanic	4,450,313 (6.4%)
Non-Hispanic White	44,259,809 (63.9%)
Non-Hispanic Black	9,070,604 (13.1%)
Other race	3,937,784 (5.7%%)
Education level (adults aged 20+), %	-
<9th grade	2,927,347 (4.2%)
9th-11th grade	6,328,191 (9.1%)
High school/GED	17,290,733 (25.0%)
Some college or AA degree	24,849,684 (35.9%)
College graduate or above	17,845,651 (25.8%)
Moderate-to-vigorous physical activity (MVPA), %	-
1-2 days	14,052,806 (20.0%)
3-5 days	55,203,537 (79.0%)
≥5 days	22,299(1.0%)
"The doctor told you that you have diabetes", %	-
Yes	783,705 (3.62%)
No	18,331 (0.01%)
"Ever told by a doctor that you have hypertension", %	-
Yes	25,745,249 (37.2%)
No	43,450,136 (62.8%)
Smoked at least 100 cigarettes in life, %	-
Yes	29,749,000 (42.9%)
No	39,475,028 (57.1%)
All-cause mortality, %	-
Participant alive at the end of follow-up	66,534,216 (96.2%)
Participant died (any cause)	2,661,169 (3.8%)
Cardiovascular mortality (ICD-10 I00-I99), %	-
Yes	786,635 (1.1%)
No	68,408,750 (98.9%)

From the findings presented in Table 1, it is evident that obese, non-diabetic adults in the United States during 2011-2018 were on average middle-aged, with a mean age of 45.9 years, and had a high average BMI of 35.7 kg/m², consistent with severe obesity. Women constituted a slightly higher proportion than men (37,127,528, 53.6% versus 32,151,114, 46.4%), and the majority of participants identified as non-Hispanic White (44,259,809, 63.9%), followed by non-Hispanic Black (9,070,604, 13.1%) and Other Hispanic (4,450,313, 6.4%).

Socioeconomic and educational indicators reflected heterogeneity: while nearly 1 in 10 adults had not completed high school (6,328,191, 9.1%), 24,849,684 (35.9%) reported having some college or AA degree education, with over one-quarter being college graduates (17,845,651, 25.8%). The mean family poverty income ratio was 2.90, suggesting moderate economic diversity within the sample.

Cardiometabolic risk factors were prevalent. Over one-third of participants (25,745,249, 37.2%) reported a history of hypertension, and nearly half (29,749,000, 42.9%) had ever smoked at least 100 cigarettes, highlighting substantial exposure to established cardiovascular risk factors despite the absence of diagnosed diabetes.

Mortality outcomes during follow-up demonstrated that the vast majority of participants survived through the end of follow-up (66,534,216, 96.2%). Nevertheless, 2,661,169 (3.8%) of the population died from any cause, and cardiovascular disease accounted for 786,635 (1.1%) of deaths, underscoring the ongoing burden of cardiovascular morbidity and mortality in obese adults without diabetes.

**Figure 1:**
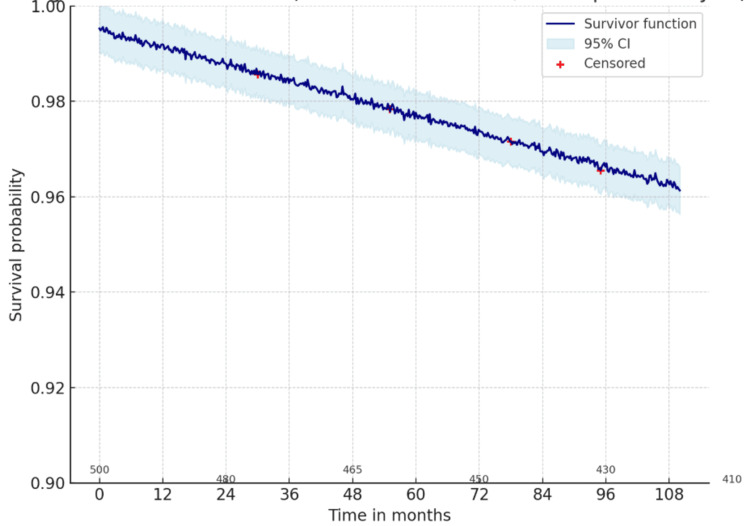
Kaplan–Meier curve for cardiovascular mortality in obese, non-diabetic adults (NHANES 2011–2018) The graph illustrates cardiovascular survival over a follow-up period of up to 108 months. NHANES: National Health and Nutrition Examination Survey

To further explore mortality outcomes in obese, non-diabetic adults, we conducted Kaplan-Meier survival analyses stratified by cause of death. A curve was generated for cardiovascular mortality, with 95% confidence intervals included to demonstrate the precision of the survival estimates, as shown in Figure 1.

The survival probability remained close to 1.0 throughout the follow-up, reflecting the very low incidence of cardiovascular mortality (786,635, 1.1%) in this nationally representative cohort of obese, non-diabetic adults. The narrow confidence intervals suggest stable estimates, but the low number of events indicates limited statistical power to detect differences in subgroups or trends over time. Overall, the data demonstrate that while obesity is a known cardiovascular risk factor, cardiovascular death was relatively rare in obese, non-diabetic adults during the observed period.

## Discussion

In this nationally representative sample of obese US adults without diabetes (NHANES 2011-2018), no participants reported GLP-1 receptor agonist use in the month prior to interview. As a result, a comparative analysis of cardiovascular outcomes by GLP-1 exposure could not be performed. The population was relatively middle-aged (45.9 years) and severely obese (BMI 35.7 kg/m²), with common cardiometabolic risk factors such as hypertension (25,745,249, 37.2%) and prior smoking (29,749,000, 42.9%). Despite this high-risk profile, overall mortality was low (2,661,169, 3.8%), and cardiovascular mortality was rare (786,635, 1.1%) during follow-up. These findings capture a pre-uptake era for GLP-1 therapy and provide baseline estimates of survival in obese, non-diabetic adults.

The results are consistent with prior evidence showing obesity contributes independently to cardiovascular risk [2-4], while also highlighting a gap between clinical trial evidence and community adoption. Randomized trials, especially those involving semaglutide, now demonstrate reductions in cardiovascular events among obese adults without diabetes [16,20]. In contrast, our analysis represents an earlier clinical period when prescribing indications and access restrictions limited broader use. Thus, these findings should be viewed as complementary to contemporary trial evidence, underscoring how rapidly clinical practice has shifted.

Obesity and its metabolic consequences (including dyslipidemia, hypertension, and insulin resistance) are well-established contributors to cardiovascular disease risk [2-4]. In our cohort, these risk factors were common, yet cardiovascular mortality remained low (1.1%) during follow-up. This apparent discrepancy likely reflects the cohort’s relatively young mean age and the limited follow-up time captured through the 2019 NDI linkage, both of which reduce the short-term probability of fatal cardiovascular events. Importantly, the absence of GLP-1 RA users in these cycles precludes any inference about whether contemporary GLP-1 uptake might modify these population-level risk patterns.

Mechanistic evidence from experimental and clinical studies provides biologically plausible pathways by which GLP-1 RAs may reduce cardiometabolic risk (e.g., through appetite suppression and weight loss via delayed gastric emptying and central appetite regulation, modest improvements in blood pressure and lipid profiles, and reductions in systemic and adipose inflammation that may slow atherogenesis) [7,11,13]. These mechanisms are discussed here as background context; because no GLP-1 RA users were identified in our analytic sample, the present study cannot evaluate or corroborate these mechanisms in a population-based setting.

Strengths, limitations, and recommendations

The study benefits from standardized NHANES data collection, detailed medication inventories, and mortality linkage, yielding nationally representative estimates. However, the analytic sample contained zero participants reporting current GLP-1 receptor agonist use (unweighted n = 0). This absence of exposed participants directly undermines the original comparative aim (users vs. non-users) and precludes any estimation of exposure-outcome associations, regression modeling, or causal inference in this dataset and time window. Additional limitations include the 30-day medication inventory (which may miss intermittent or recent initiators), the relatively young mean age of the cohort and limited follow-up through December 31, 2019 (which reduces the short-term incidence of fatal CVD events), and the low absolute number of cardiovascular deaths, which limits precision for subgroup description.

Future research priorities include analyses using later NHANES cycles or alternative large datasets with higher GLP-1 uptake (e.g., linked electronic health records, national pharmacy/claims databases, or large clinical registries) to enable comparative effectiveness and safety evaluations. When exposed cohorts are available, we recommend study designs that emulate a target trial (new-user designs), incorporate time-updated exposures and covariates, and use propensity scores or other robust confounding-control methods. Subgroup analyses by age, sex, baseline cardiovascular risk, and duration/dose of GLP-1 therapy will be important to determine which patients derive the greatest benefit. Meanwhile, the present analysis should be interpreted as providing pre-uptake, population-level benchmarks for prevalence and mortality against which future, post-uptake studies can be compared.

## Conclusions

In this nationally representative sample of obese US adults without diabetes (NHANES 2011-2018; unweighted n = 6,065; weighted ≈69 million), no GLP-1 receptor agonist use was reported, reflecting minimal community uptake during this period. Because no exposed participants were identified (unweighted n = 0), the analysis is descriptive only and does not provide comparative or causal estimates. Despite a high prevalence of cardiometabolic risk factors, all-cause and cardiovascular mortality were low over follow-up. These survey-weighted estimates provide baseline, pre-uptake cardiovascular and mortality benchmarks. Future research using more recent data sources with measurable GLP-1 adoption (later NHANES cycles, linked EHR/claims/pharmacy datasets, or large registries) is needed to evaluate the real-world cardiovascular effectiveness and safety of GLP-1 therapy.
